# New Polymeric Films with Antibacterial Activity Obtained by UV-induced Copolymerization of Acryloyloxyalkyltriethylammonium Salts with 2-Hydroxyethyl Methacrylate

**DOI:** 10.3390/ijms20112696

**Published:** 2019-05-31

**Authors:** Francesco Galiano, Raffaella Mancuso, Maria Grazia Guzzo, Fabrizio Lucente, Ephraim Gukelberger, Maria Adele Losso, Alberto Figoli, Jan Hoinkis, Bartolo Gabriele

**Affiliations:** 1 Institute on Membrane Technologies (ITM-CNR), Via Pietro Bucci 17/C, 87036 Arcavacata di Rende (CS), Italy; f.galiano@itm.cnr.it; 2 Laboratory of Industrial and Synthetic Organic Chemistry (LISOC), Department of Chemistry and Chemical Technologies, University of Calabria, 87036 Arcavacata di Rende (CS), Italy; raffaella.mancuso@unical.it (R.M.); ephraim.gukelberger@hs-karlsruhe.de (E.G.); 3 Department of Biology, Ecology, and Earth Sciences (DiBEST), University of Calabria, 87036 Arcavacata di Rende (CS), Italy; mariagraziaguzzo22@gmail.com (M.G.G.); fabrizio.lucente93@gmail.com (F.L.); 4 University of Applied Sciences Karlsruhe, Center of Applied Research (CAR), Moltkestraße 30, 76133 Karlsruhe, Germany; jan.hoinkis@hs-karlsruhe.de

**Keywords:** acrylates, antibacterial activity, copolymerization, polymeric films, polymerizable quaternary ammonium salts, quaternary ammonium salts, UV-induced polymerization

## Abstract

New polymeric films with antibacterial activity have been prepared, by simple UV-induced copolymerization of readily available ω-(acryloyloxy)-*N*,*N*,*N*-triethylalcan-1-aminium bromides (or acryloyloxyalkyltriethylammonium bromides, AATEABs) with commercially available 2-hydroxyethyl methacrylate (HEMA), at different relative amounts. In particular, the antibacterial activity of polymeric films derived from 11-(acryloyloxy)-*N*,*N*,*N*-triethylundecan-1-aminium bromide (or acryloyloxyundecyltriethylammonium bromide, AUTEAB; bearing a C-11 alkyl chain linker between the acrylate polymerization function and the quaternary ammonium moiety) and 12-(acryloyloxy)-*N*,*N*,*N*-triethyldodecan-1-aminium bromide (or acryloyloxydodecyltriethylammonium bromide, ADTEB, bearing a C-12 alkyl chain linker) has been assessed against Gram-negative *Escherichia Coli* and Gram-positive *Staphylococcus aureus* cells. The results obtained have shown a clear concentration-dependent activity against both bacterial strains, the films obtained from homopolymerization of pure AUTEAB and ADTEAB being the most effective. Moreover, ADTEAB-based films showed a higher antibacterial activity with respect to the AUTEAB-based ones. Interestingly, however, both types of films presented a significant activity not only toward Gram-positive *S. aureus*, but also toward Gram-negative *E. Coli* cells.

## 1. Introduction

The importance of developing new antimicrobial systems is becoming more and more important, owing to the well-known increasing phenomena of resistance to antibiotics associated with an augmented virulence of several pathogenic microbial species [1,2,3,4,5]. In particular, antimicrobial polymers have recently attracted high interest, in view of their significant, efficient, and broad-spectrum activity against resistant microorganisms [6,7,8,9,10,11,12,13,14]. Moreover, antimicrobial polymers can find extensive applications in several applicative fields [15], including health care and biomedical applications [16,17,18,19,20], food conservation and packaging [21,22,23,24], and industry (membrane [25,26,27,28] and textile industry [29,30,31,32], in particular).

It is well known that quaternary ammonium salts (QASs) present a strong antimicrobial activity (against fungi, bacteria, and viruses, in particular) [33,34,35,36,37,38,39,40], which is mainly related to their ability to promote an ionic exchange between the membrane cell and the positively charged group, thus leading to the loss of membrane integrity and cell death [41,42]. Polymerizable quaternary ammonium salts (PQASs) are a particularly interesting subclass of QASs, which is characterized by the presence, besides the quaternary group, of a suitable polymerizable function. This may allow their incorporation into a polymeric framework by means of copolymerization techniques, thus leading to polymeric materials with antimicrobial properties [43,44,45].

**Scheme 1:**
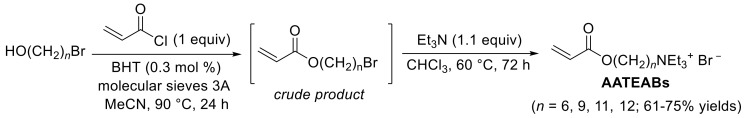
Synthesis of acryloyloxyalkyltriethylammonium bromides (AATEABs) [46].

In this field, we recently reported a novel and practical synthetic approach to a particularly interesting class of polymerizable quaternary ammonium salts (PQASs), which are ω-(acryloyloxy)-*N*,*N*,*N*-triethylalcan-1-aminium bromides (or acryloyloxyalkyltriethylammonium bromides, AATEABs), as shown in Scheme 1 [46].

These compounds are characterized by the presence, on one hand, of a quaternary ammonium moiety, which confers them a significant antimicrobial activity, and, on the other hand, of an acryloyloxy function, which make these compounds easily polymerizable either by radical- [47,48] or UV-induced [49] polymerization. The two active terminal moieties are distanced through a suitable alkyl chain linker. We previously assessed the antimicrobial activity of the newly synthetized AATEABs against several Gram-positive and Gram-negative bacteria and yeast strains [46]. The results obtained showed that the AATEABs bearing a C-11 and a C-12 alkyl chain linker (11-(acryloyloxy)-*N*,*N*,*N*-triethylundecan-1-aminium bromide or acryloyloxyundecyltriethylammonium bromide, AUTEAB, and 12-(acryloyloxy)-*N*,*N*,*N*-triethyldodecan-1-aminium bromide (or acryloyloxydodecyltriethylammonium bromide, ADTEB, respectively) were the most active, in particular, against Gram-positive bacteria *Staphylococcus aureus* and *Streptococcus pyogenes* [46]. The higher bioactivity of AUTEAB and ADTEAB with respect to the other derivatives with shorter alkyl chain linkers was also recently theoretically interpreted by ab initio modeling calculations [50].

Considering the promising antibacterial activity of AUTEAB and ADTEAB [46,50], and the possibility to easily copolymerize them for obtaining new antibacterial materials [47,48,49], in this work we have studied the development of new polymeric films chemically incorporating these PQASs, for potential applications in biomedical, food packaging and textile field. In particular, we have prepared polymeric films based on the UV-induced copolymerization of AUTEAB as well as ADTEAB with commercially available 2-hydroxyethyl methacrylate (HEMA), at different relative amounts. The new films thus obtained were assessed for their antibacterial activity, at different concentrations, towards the two bacteria strains *E. coli* and *S. aureus*. The possibility to copolymerize antimicrobial AUTEAB and ADTEAB with HEMA is of particular interest, considering that the homopolymer obtained by polymerization of HEMA (pHEMA) is very well appreciated for its transparency and biocompatibility, properties that make pHEMA an ideal candidate for the production of contact lenses and other products in the biomedical field [51].

## 2. Results and Discussion

**Figure 1:**
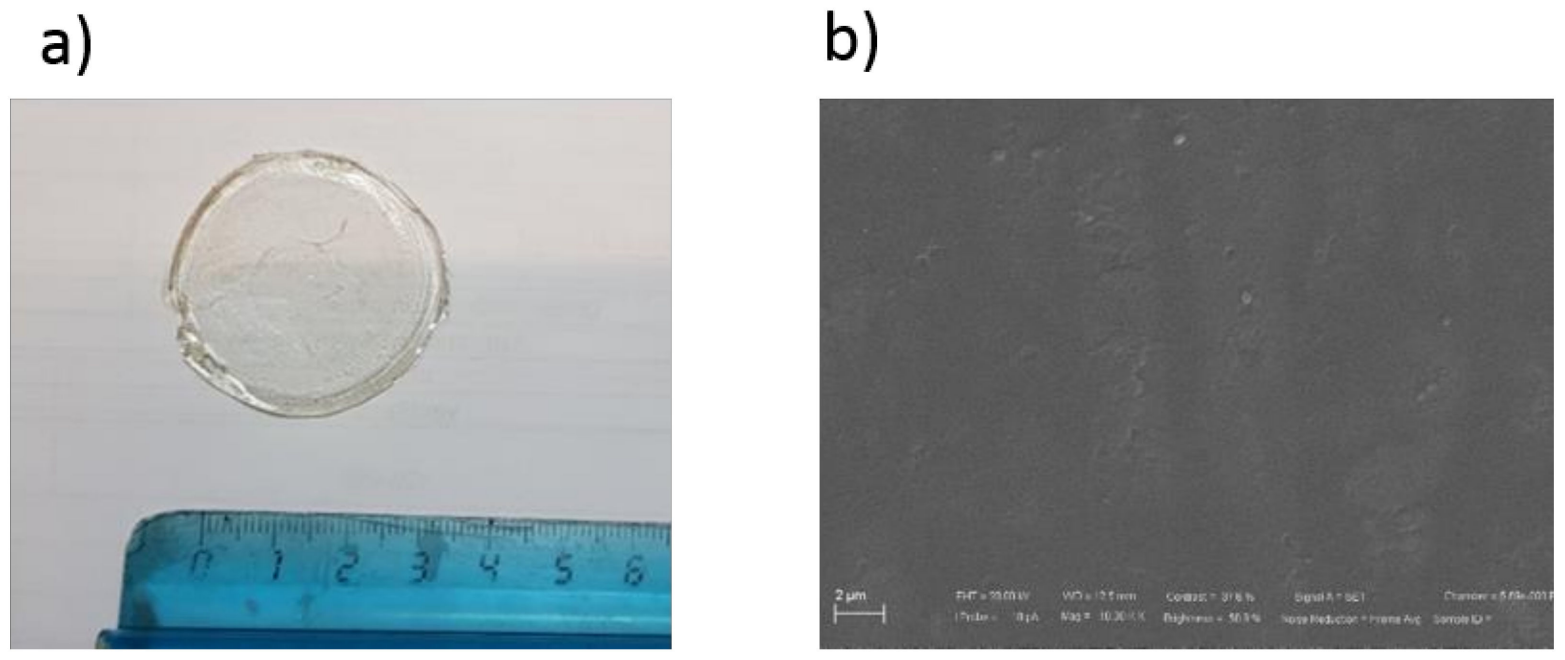
Image (**a**) and SEM picture (**b**) of a typical film obtained by copolymerization of acryloyloxyundecyltriethylammonium bromide (AUTEAB) with 2-hydroxyethyl methacrylate (HEMA).

The new antimicrobial polymeric films developed in this work were obtained by UV-induced copolymerizazion of the PQASs AUTEAB or ADTEAB with commercially available HEMA, at different relative amounts. In particular, a proper amount of the PQAS (15.0, 35.0, 50.0 and 100 wt%) and HEMA (85, 65, 50, and 0 wt%, respectively) were mixed until a transparent solution was obtained. A small amount of the UV initiator 2,2-dimethoxy-2-phenylacetophenone (DMPA) was then added, and the mixture was poured in a glass petri dish and exposed to 500 W UV light irradiation (lamp emission from 180 nm to visible light). Polymerization was quite fast, and after 10 min transparent films were obtained (Figure 1a), which were detached form the petri dish in water and washed in water overnight, ready to be used for the subsequent antibacterial tests. As shown in scanning electron microscope (SEM) picture (Figure 1b), the film surface appeared characterized by a uniform, dense, and compact morphology. The morphology was practically the same for all the prepared films. The films presented an overall thickness of about 0.432 mm.

**Figure 2:**
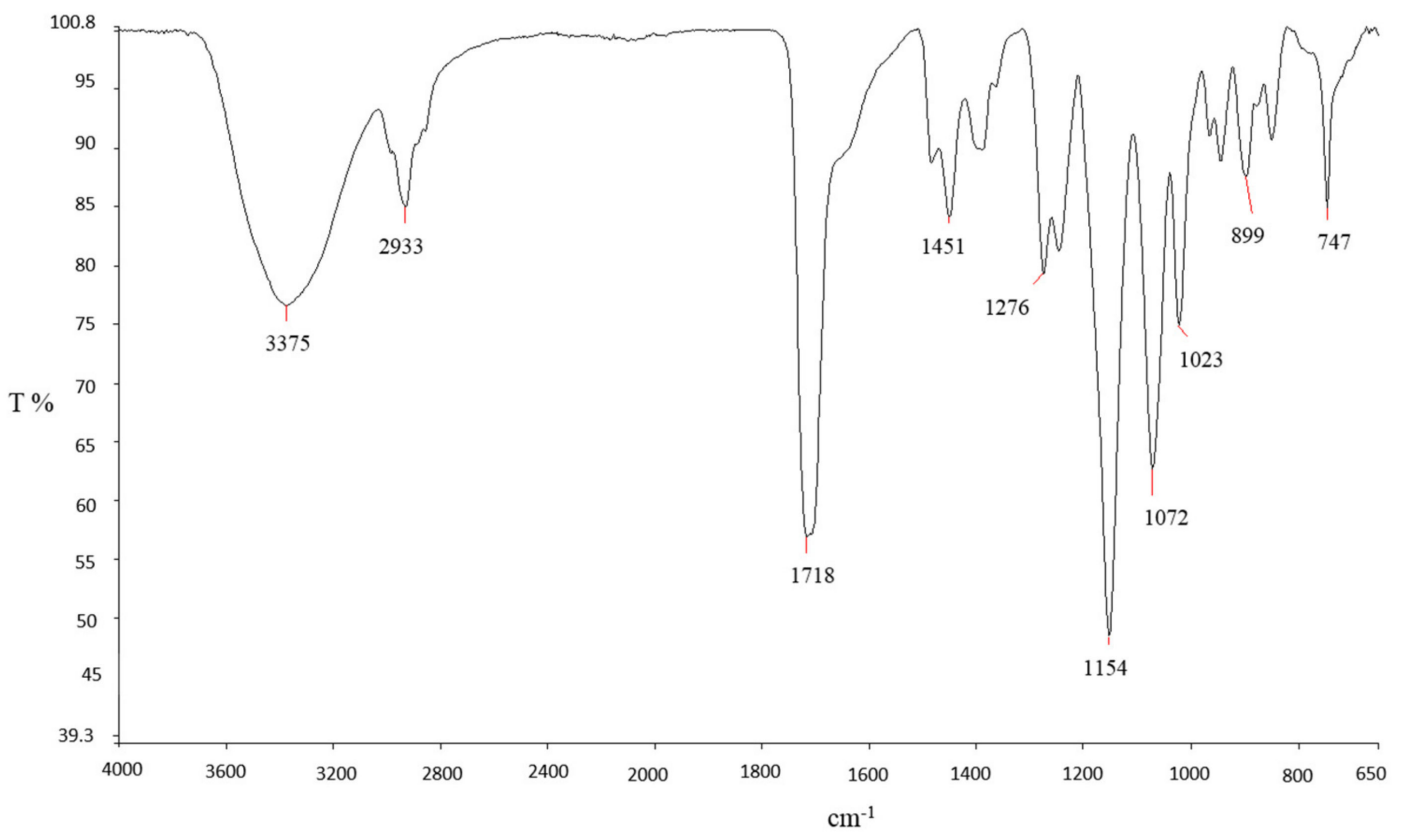
FT-IR spectrum of the polymeric film prepared with 15 wt% ADTEAB and 85 wt% HEMA.

**Figure 3:**
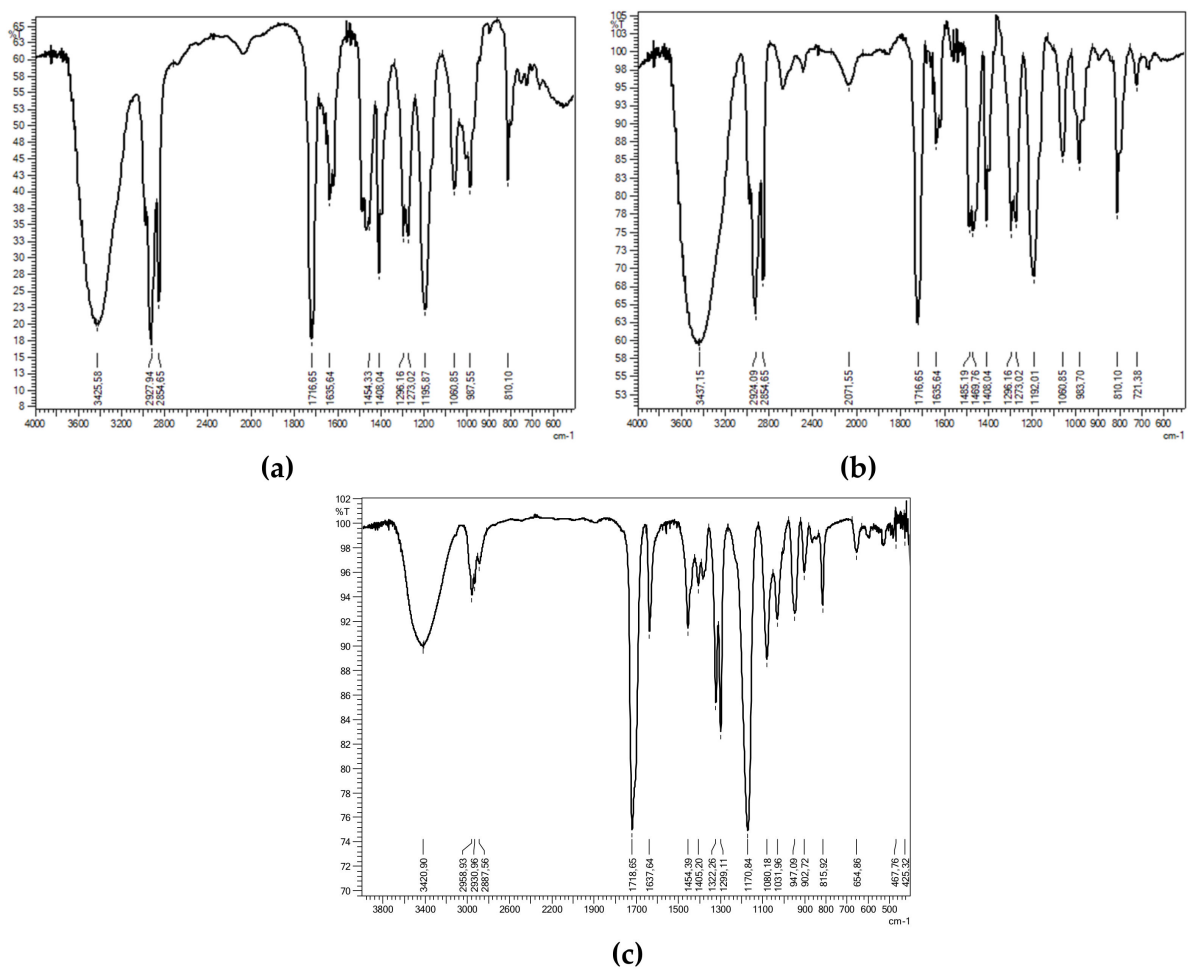
FT-IR spectra of AUTEAB (**a**), ADTEAB (**b**), and HEMA (**c**).

An exemplificative Fourier transform infrared spectroscopy (FT-IR) spectrum of a film prepared with 15.0 wt% of ADTEAB and HEMA is reported in Figure 2. The wide and intense band at 3375 cm^−1^ can be assigned to the O‒H stretching vibrations of pHEMA [52]. At 1718 cm^−1^, it can be observed the stretching vibrations of the carbonyl C=O group (from both ADTEAB and pHEMA), which is generally found in the region of 1650–1800 cm^−1^ [53]. The region between 2900 and 3000 cm^−1^ is associated with the symmetric and anti-symmetric C‒H vibrations of CH_2_ and CH_3_ groups of ADTEAB and pHEMA [52]. For comparison, the FT-IR spectra of AUEAB, ADTEAB, and HEMA are shown in Figure 3.

**Figure 4:**
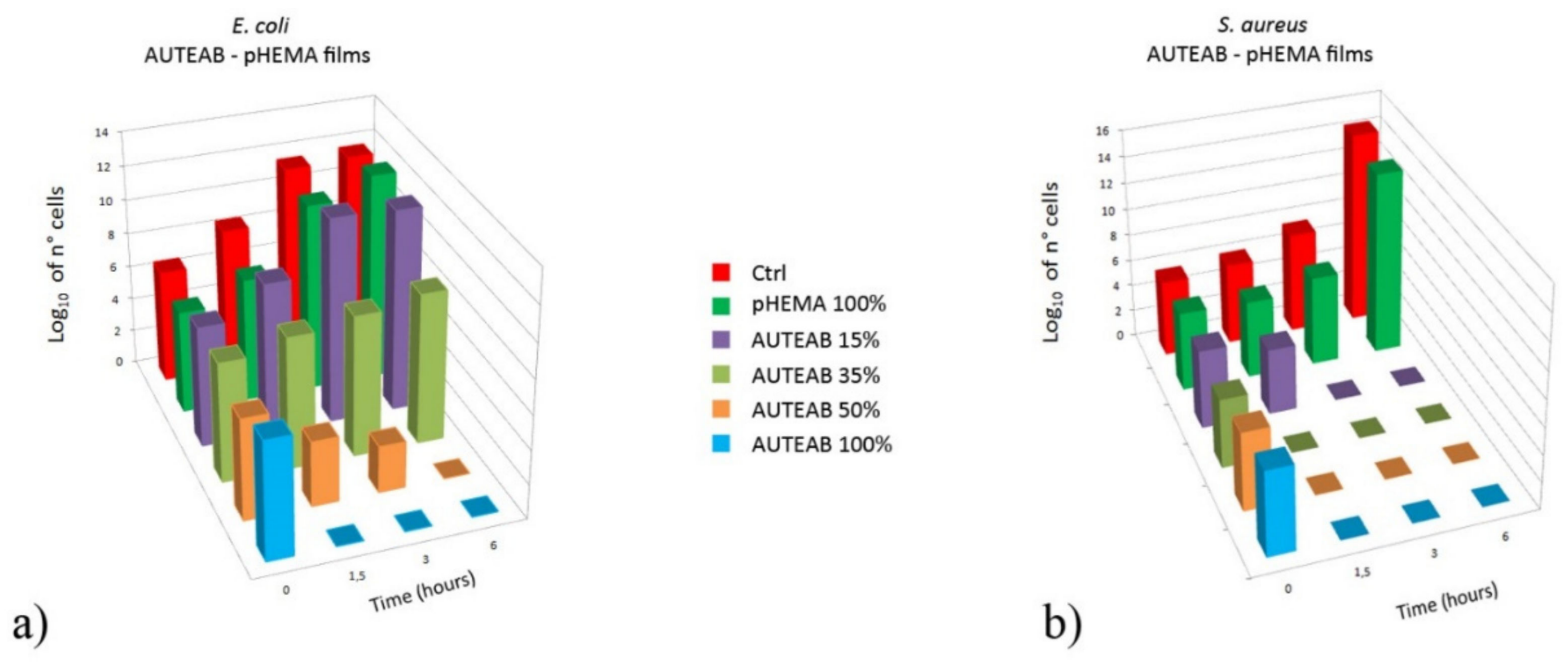
Comparison of viable cell number of *E. coli* TG1 (**a**) and *S. aureus* (**b**) as a function of time in the presence of control (no film, red histogram), blank reference (pHEMA, dark green histogram), 15% AUTEAB film (purple histogram), 35% AUTEAB film (light green histogram), 50% AUTEAB film (orange histogram) and 100% AUTEAB film (light blue histogram). Bacteria cells were grown in Luria-Bertani broth and the cell number was determined by surface spread plate technique, as described in the Materials and Methods Section.

The antibacterial efficacy of the AUTEAB-HEMA and ADTEAB-HEMA polymeric films thus obtained, and of pHEMA as blank reference, was assessed on the basis of cell viability of *E. coli* TG1 (Gram-negative) and *Staphilococcus aureus* (Gram-positive) cultures, after being in contact with the films at different times (0, 1.5, 3, and 6 h). Figure 4a shows a comparison between the cell viability of *E. coli* TG1 in presence of AUTEAB-HEMA films at different incubation times. No loss of viable bacteria was detected in the cells control (no film exposure) and in the blank reference pHEMA (histograms in red and dark green, respectively). On the other hand, a clear concentration-dependent effect was observed with AUTEAB-HEMA polymeric films. While no antibacterial activity was obtained after 6 h with the 15% AUTEAB film (purple histogram, Figure 4a), a bacteriostatic effect was evident with the 35% AUTEAB film (light green histogram, Figure 4a), and a bactericidal activity with the 50% AUTEAB film (orange histogram, Figure 4a). As expected, the bactericidal effect was dramatic in the case of the homopolymeric film obtained from AUTEAB only (light blue histogram, Figure 4a). In fact, in this latter case, no viable cells could be detected after 1.5 h contact (light blue histogram, Figure 4a), while with the 50% AUTEAB film at the same incubation time, we observed a reduction of cell viability of about two orders of magnitude, and cell viability reached 0 only after 6 h of incubation (orange histogram, Figure 4a). The antimicrobial activity against the Gram-positive *S. aureus,* shown in Figure 4b, was more pronounced, as the bactericidal effect was reached either after only 1.5 h contact with the 50% AUTEAB film (orange histogram, Figure 4b) or after 3 h with the 15% AUTEAB film (purple histogram, Figure 4b).

**Figure 5:**
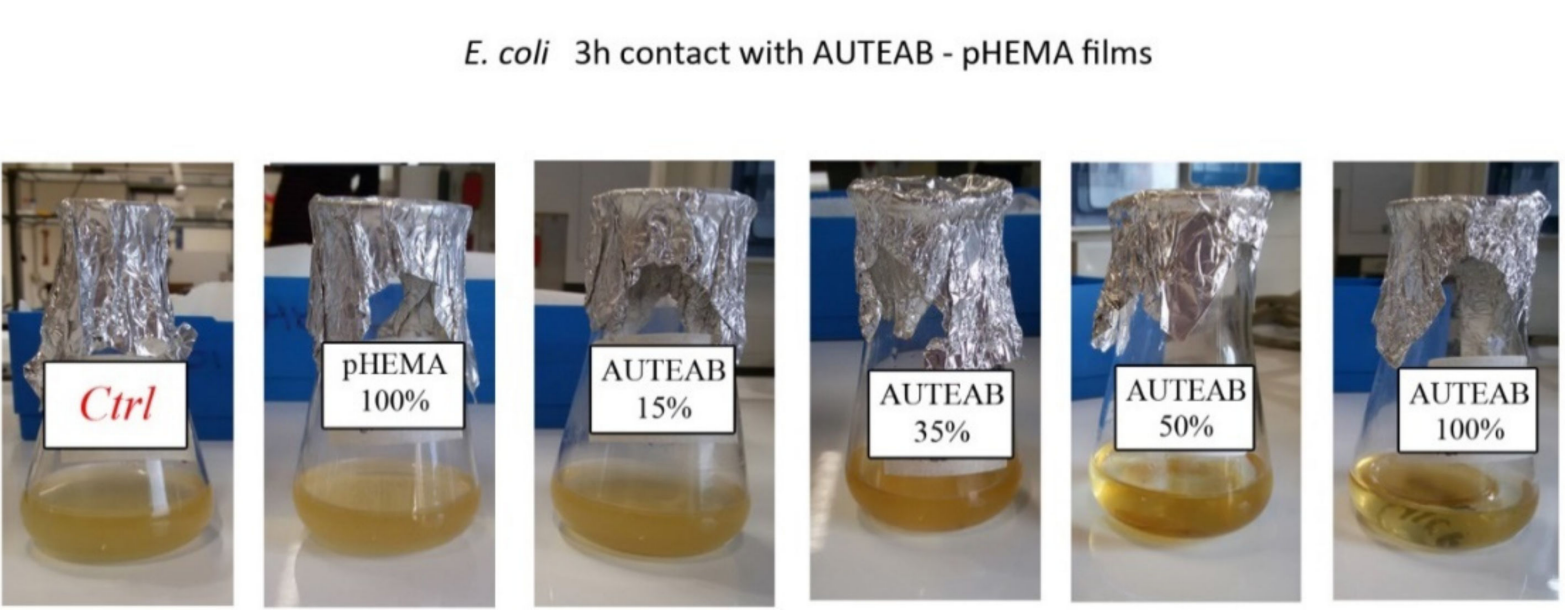
*E. coli* cultures maintained in contact with AUTEAB-HEMA polymeric films obtained with 15, 35, 50, and 100% AUTEAB. Control (no film exposure) and blank reference (pHEMA) are also shown for comparison.

Figure 5 shows the reduction of turbidity of the *E. coli* cultures in presence of AUTEAB-based films after 3 h contact with the film. It is evident that the degree of cell population in the medium (as evidenced by the culture turbidity) decreases by increasing the % of AUTAB in the AUTEAB-HEMA polymeric film. With the film obtained by homopolymerization of AUTEAB, the mixture is clear, confirming that no cell population is present.

It is known that the antibacterial mechanism of QASs is mainly related to their strong interaction with the cell membrane which causes its disorganization. This leads to the degradation of nucleic acids and proteins with consequent lysis of the bacterial cell wall by autolytic enzymes [41,42]. Usually, QASs present a significant different antibacterial efficiency toward Gram-positive and Gram-negative bacteria, due to the additional outer membrane in the Gram-negative bacteria, which is absent in the Gram-positive ones [52]. For this reason, the multilayer structure of the membrane makes the Gram-negative bacteria more resistant toward the access and the internalization in the cytoplasm of QASs. Our results, obtained with AUTEAB-HEMA polymeric films, while confirming a higher antimicrobial activity against the Gram-positive *S. aureus* with respect to the Gram-negative *E. coli* (compare Figure 4a with Figure 4b), also demonstrate that a significant concentration-dependent antimicrobial effect is exerted on the latter, with a bactericidal effect being observed after 6 h with the 50% AUTEAB film and after only 1.5 h with 100% AUTEAB film (Figure 4a).

We also prepared and tested polymeric films using acryloyloxydodecyltriethylammonium bromide (ADTEAB), bearing a C-12 rather than a C-11 alkyl linker. This derivative, in fact, presented a higher antimicrobial activity compared to the C-11 compound (AUTEAB) [46], which was also in agreement with ab initio modeling calculations [50]. In this latter theoretical work, it was demonstrated that the increase in the antimicrobial activity of QAS molecules could be mainly attributed to their “aspect ratio” [50]. QAS with a longer alkyl chain bonded to nitrogen, in fact, exhibited a lower aspect ratio resulting in a higher shielding effect on the quaternary ammonium group [50], which is known to cause a higher antimicrobial activity [53]. Interestingly, the “shielding” effect increases with the alkyl chain length but only up to a certain limit. For example, He et al. [54] found that the optimum alkyl chain length for the antimicrobial effect against *S. mutans* cells was between C-11 and C-16, while the effect tended to lower with longer alkyl chains.

**Figure 6:**
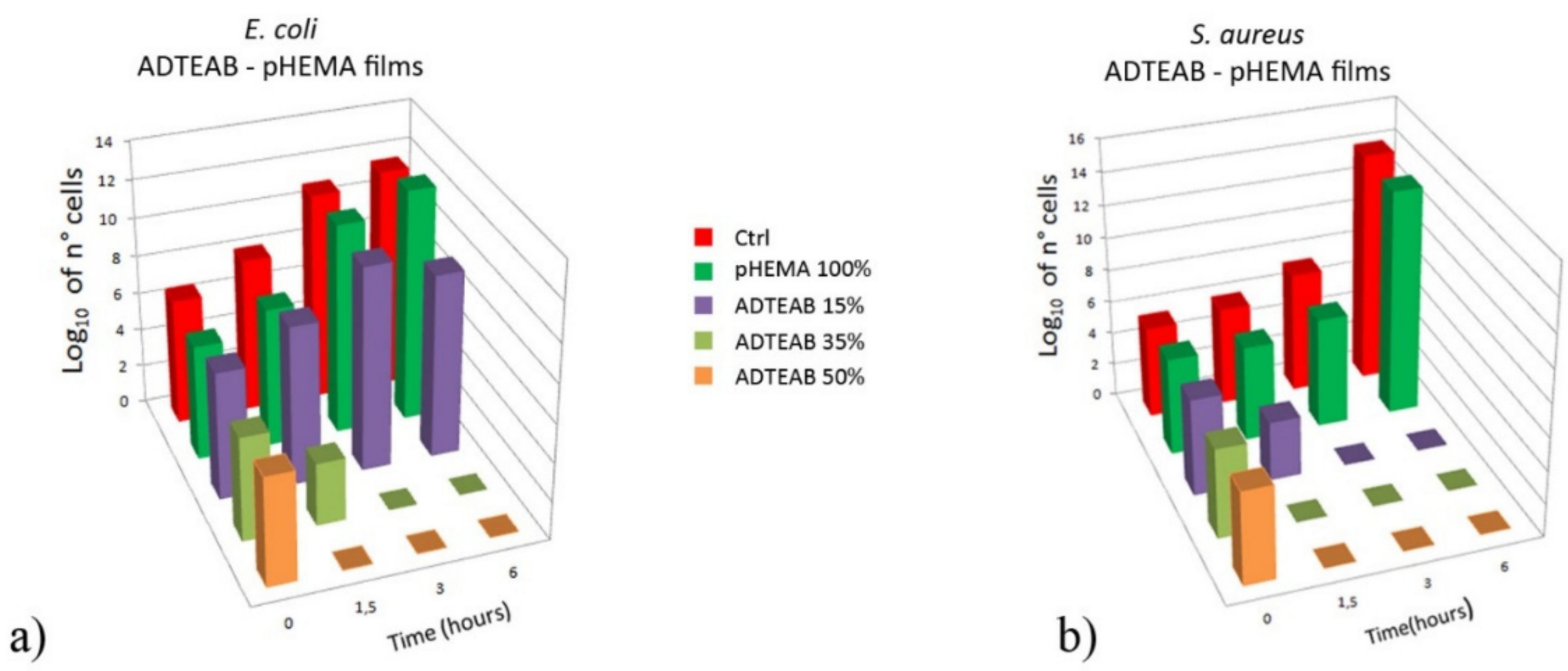
Comparison of viable cell number of *E. coli* TG1 (**a**) and *S. aureus* (**b**) as a function of time in the presence of control (no film, red histogram), blank reference (pHEMA, dark green histogram), 15% ADTEAB film (purple histogram), 35% ADTEAB film (light green histogram) and 50% ADTEAB film (orange histogram). Bacteria cells were grown in Luria-Bertani broth and the cell number was determined by surface spread plate technique, as described in the Materials and Methods Section.

The results obtained with ADTEAB-HEMA polymeric films are shown in Figure 6, together with the control (no film exposure) and the blank reference pHEMA. As expected (Figure 6a), no loss of viable *E. coli* cells was detected in the cells control and with pHEMA (histograms in red and dark green, respectively). Additionally, no activity was observed with the 15% ADTEAB film (purple histogram, Figure 6a). On the other hand, an evident bactericidal effect was obtained with 35 and 50% ADTEAB film after 3 and 1.5 h contact, respectively (light green and orange histograms, Figure 6a). As seen for AUTEAB-based films (Figure 4), the antimicrobial activity of ADTEAB-based films on the Gram-positive *S. aureus* was higher (Figure 6b). In fact, the bactericidal effect was reached after either 1.5 h with 35% and 50% ADTEAB film (Figure 6b, light green and orange histograms, respectively). Moreover, a viable cells decrease, of about 2 order of magnitude, was already seen with 15% ADTEAB after 1.5 h and no viable cells were detected at 3 h contact (Figure 6b, purple histogram), while no activity was observed with the same film against *E. coli* (purple histogram, Figure 6a). These results also confirmed a higher antimicrobial activity for ADTEAB-based films (Figure 6) in comparison to the ones prepared with AUTEAB (Figure 4). Rather interestingly, this was particularly evident in the Gram-negative *E. coli*.

## 3. Materials and Methods

### 3.1. Preparation of Polymeric Films

Antimicrobial films were prepared by mixing a proper amount of AUTEAB or ADTEAB (prepared as we already reported [46]) (15.0, 35.0, 50.0 and 100 wt%) with HEMA (purchased by Sigma-Aldrich Italia, Milan, Italy) (85, 65, 50, and 0 wt%, respectively). Films containing 100 wt% of antimicrobial agent were prepared by dissolving AUTEAB in water with a ratio of 75:25 AUTEAB/water. Solid films containing 100 wt% ADTEAB could not be prepared due to the high fragility of the polymerized material. The mixtures (1 g) were, then, stirred for 1 h until complete dissolution. The UV initiator 2,2-dimethoxy-2-phenylacetophenone DMPA (2,2-dimethoxy-1,2-diphenylethan-1-one; purchased by Sigma-Aldrich Italia, Milan, Italy) (0.6 wt% with respect to the total amount of the mixture) was then added. After 1 h stirring, the solutions were poured in a glass petri dish (3 cm in diameter) and exposed for polymerization to UV light irradiation (lamp emission from 180 nm to visible light, 500W; purchased from Helios Italquarz, Cambiago, Milan, Italy) for 10 min. The polymerized films were then detached form the petri dish in water and washed in water overnight. A blank film, not containing any antimicrobial agent, was also prepared through the polymerization of pure HEMA with 0.6 wt% of DMPA. Table 1 shows the composition of the prepared films.

Fourier transform infrared spectroscopy (FT-IR) analysis was performed by using a Perkin Elmer Instrument (New York, NY, USA) in the range 4000–650 cm^−1^; for the polymeric films, Attenuated Total Reflection (ATR) mode was used, while for HEMA, AUTEB and ADTEAB, KBr pellets were prepared. SEM image was acquired by means of Zeiss-EVO MA10 (thermal emission tungsten firing unit equipped with a secondary electron detector) instrument using an Electron High Tension (EHT) of 20 kV and with a probe current of 18 pA. Prior to analyses, the sample was coated with a thin layer of gold (sputter current of 20 mA and a sputter time of 240 s) using a sputter coater machine (Quorum Q150 RS) in order to make the sample conductive.

### 3.2. Microorganisms and General Growth Conditions

*E. coli* TG1 and *Staphilococcus aureus*, kindly provided by Prof. Michele Galluccio (Department of Ecology, Biology and Earth Sciences, University of Calabria, Rende, Italy), were selected as Gram-negative and Gram-positive bacteria, respectively. The two strains were grown aerobically, at 37 °C and 200 rpm in a thermostatic orbital shaking incubator (Sanyo Gallenkamp IOX400.XX1.C, Analitica De Mori, Milan, Italy), in sterile Luria Bertani (LB) broth, a rich growth medium (containing sodium chloride 5 g/L; yeast extract 5 g/L; trypton, 10 g/L at pH = 6.8). Oxoid Italia (Rodano, Milan, Italy) supplied the powders for medium preparation.

### 3.3. Antibacterial Assessment of Polymeric Films

An overnight culture of *E. coli* TG1 or *S. aureus* was diluted 1:100 (*v*/*v*) in 20 mL of fresh LB liquid medium to restart the cell cycle and incubated for circa 3 h at 37 °C until the exponential growth phase was reached. Then, the culture was diluted 1:10 (*v*/*v*) in 20 mL of LB liquid medium, in an Erlenmeyer flask, to reach a final density of circa 10^6^ CFU/mL with an optical density, at 600 nm (OD_600_), of about 0.06 [55]. This value was chosen on the basis of our preliminary data on growth curves of *E. coli* TG1 performed to correlate OD measurements and number of cells by plate counting. Finally, each different preparation of film was immersed in the bacterial suspension and shaken at 37 °C for 6 h. A same assay procedure was used for bacterial suspensions without films used as control.

The effect of antimicrobial films at different concentration on the bacterial growth has been assessed after 0, 1.5, 3, and 6 h, by OD_600_ and cell viability assays. To calculate the Colony Forming Unit (CFU) at the predetermined time, 100 μL of bacteria culture was taken from the flasks with and without film and decimal serial dilutions in LB were performed. 50 μL of the diluted sample were then spread onto sterile LB agar plates (LB broth with the addition of 16 g/L of agar). After incubation of the plates at 37 °C for 20–24 h, the number of viable cells (colonies) was counted manually to get the corresponding concentration of living bacteria. The log of N (cell number) was calculated using the formula: CFU/mL = n^o^ of colonies × dilution factor/volume of culture spread. CFU for every time was calculated on the average of three different dilutions.

## 4. Conclusions

In conclusion, we have developed novel polymeric films based on UV-induced copolymerization of some readily available polymerizable quaternary ammonium salts (QASs), in particular, 11-(acryloyloxy)-*N*,*N*,*N*-triethylundecan-1-aminium bromide or acryloyloxyundecyltriethylammonium bromide, AUTEAB, and 12-(acryloyloxy)-*N*,*N*,*N*-triethyldodecan-1-aminium bromide or acryloyloxydodecyltriethylammonium bromide, ADTEB, with commercially available 2-hydroxyethyl methacrylate (HEMA). The antibacterial tests, conducted on typical Gram-negative (*E. Coli*) and Gram-positive (*S. aureus*) strains, have confirmed a significant antibacterial activity, not only against the Gram-positive cells, but also on the Gram-negative ones (which are known to be usually much more resistant toward QASs), although the activity was higher in the first case. Moreover, the results obtained have shown that the activity depended on the QAS concentration in the film and that is was higher for the films obtained from ADTEAB with respect to AUTEAB.

The possible application of the newly prepared films in various fields (biomedical, textile, and membrane technology, in particular) is underway in our laboratories and the results will be reported in due course.

## Figures and Tables

**Table 1:** Relative amounts of the components used for preparing the polymeric films.

Film	AATEAB (wt%)	HEMA (wt%)	Water (wt%)
AUTEAB 15%	AUTEAB (15)	(85)	(0)
AUTEAB 35%	AUTEAB (35)	(65)	(0)
AUTEAB 50%	AUTEAB (50)	(50)	(0)
AUTEAB 100%	AUTEAB (75)	(0)	(25) ^1^
ADTEAB 15%	ADTEAB (15)	(85)	(0)
ADTEAB 35%	ADTEAB (35)	(65)	(0)
ADTEAB 50%	ADTEAB (50)	(50)	(0)
pHEMA	(0)	(100)	(0)

^1^ The final polymeric film contained only polymerized AUTEAB, since water evaporated completely during the polymerization process.

## Abbreviations

AATEABs	acryloyloxydodecyltriethylammonium bromide (ω-(acryloyloxy)-*N*,*N*,*N*-triethylalcan-1-aminium bromides)
ADTEAB	acryloyloxydodecyltriethylammonium bromide (12-(acryloyloxy)-*N*,*N*,*N*-triethylundecan-1-aminium bromide)
AUTEAB	acryloyloxyundecyltriethylammonium bromide(11-(acryloyloxy)-*N*,*N*,*N*-triethyldodecan-1-aminium bromide)
CFU	colony-forming unit
DMPA	2,2-dimethoxy-2-phenylacetophenone (2,2-dimethoxy-1,2-diphenylethan-1-one)
HEMA	2-hydroxyethyl methacrylate
LB	Luria-Bertani
OD	optical density
pHEMA	poly-2-hydroxyethyl methacrylate
PQASs	polymerizable quaternary ammonium salts
QASs	quaternary ammonium salts

## References

[1] Almakki A., Jumas-Bilak E., Marchandin H., Licznar-Fajardo P. (2019). Antibiotic resistance in urban runoff. Sci. Total Environ..

[2] Canica M., Manageiro V., Abriouel H., Moran-Gilad J., Franz C.M.A.P. (2019). Antibiotic resistance in foodborne bacteria. Trends Food Sci. Technol..

[3] Li R., Jay J.A., Stenstrom M.K. (2019). Fate of antibiotic resistance genes and antibiotic-resistant bacteria in water resource recovery facilities. Water Environ. Res..

[4] Peterson E., Kaur P. (2018). Antibiotic resistance mechanisms in bacteria: Relationships between resistance determinants of antibiotic producers, environmental Bacteria, and clinical pathogens. Front. Microbiol..

[5] Babakhani S., Oloomi M. (2018). Transposons: The agents of antibiotic resistance in bacteria. J. Basic Microbiol..

[6] Xing H., Lu M., Yang T., Liu H., Sun Y., Zhao X., Xu H., Yang L., Ding P. (2019). Structure-function relationships of nonviral gene vectors: Lessons from antimicrobial polymers. Acta Biomater..

[7] Munoz-Bonilla A., Echeverria C., Sonseca A., Arrieta M.P., Fernandez-Garcia M. (2019). Bio-based polymers with antimicrobial properties towards sustainable development. Materials.

[8] Konai M.M., Bhattacharjee B., Ghosh S., Haldar J. (2018). Recent progress in polymer research to tackle infections and antimicrobial resistance. Biomacromolecules.

[9] Ergene C., Yasuhara K., Palermo E.F. (2018). Biomimetic antimicrobial polymers: Recent advances in molecular design. Polym. Chem..

[10] Al-Jumaili A., Kumar A., Bazaka K., Jacon M.V. (2018). Plant secondary metabolite-derived polymers: A potential approach to develop antimicrobial films. Polymers.

[11] Ergene C., Palermo E.F. (2018). Antimicrobial synthetic polymers: An update on structure-activity relationships. Curr. Pharm. Des..

[12] Yang Y., Cai Z., Huang Z., Tang X., Zhang X. (2018). Antimicrobial cationic polymers: From structural design to functional control. Polym. J..

[13] Lam S.J., Wong E.H.H., Boyer C., Qiao G.G. (2018). Antimicrobial polymeric nanoparticles. Progr. Polym. Sci..

[14] Alvarez-Paino M., Munoz-Bonilla A., Fernandez-Garcia M. (2017). Antimicrobial polymers in the nano-world. Nanomaterials.

[15] Huang K.-S., Yang C.-H., Huang S.-L., Chan C.-Y., Lu Y.-Y., Lin Y.-S. (2016). Recent advances in antimicrobial polymers: A mini-review. Int. J. Mol. Sci..

[16] Gonzalez-Henriquez C.M., Sarabia-Vallejos M.A., Rodriguez Hernandez J. (2019). Antimicrobial polymers for additive manufacturing. Int. J. Mol. Sci..

[17] Feldman D. (2018). Polymer nanocomposites for tissue engineering, antimicrobials and drug delivery. Biointerface Res. Appl. Chem..

[18] Gahruie H.H., Niakousari M. (2017). Antioxidant, antimicrobial, cell viability and enzymatic inhibitory of antioxidant polymers as biological macromolecules. Int. J. Biol. Part A Macromol..

[19] Hartlieb M., Williams E.G.L., Kuroki A., Perrier S., Locock K.E.S. (2017). Antimicrobial polymers: Mimicking amino acid functionality, sequence control and three-dimensional structure of host-defense peptides. Curr. Med. Chem..

[20] Wo Y., Brisbois E.J., Bartlett R.H., Meyerhoff M.E. (2016). Recent advances in thromboresistant and antimicrobial polymers for biomedical applications: Just say yes to nitric oxide (NO). Biomater. Sci..

[21] Mujtaba M., Morsi R.E., Kerch G., Elsabee M.Z., Kaya M., Labidi J., Khawar K.M. (2019). Current advancements in chitosan-based film production for food technology; A review. Int. J. Biol. Macromol..

[22] Vasile C. (2018). Polymeric nanocomposites and nanocoatings for food packaging: A review. Materials.

[23] Santos J.C.P., Sousa R.C.S., Otoni C.G., Moraes A.R.F., Souza V.G.L., Medeiros E.A.A., Espita P.J.P., Pires A.C.S., Coimbra J.S.R., Soares N.F.F. (2018). Nisin and other antimicrobial peptides: Production, mechanisms of action, and application in active food packaging. Innov. Food Sci. Emerg. Technol..

[24] Galie S., Garcia-Gutierrez C., Miguelez E.M., Villar C.J., Lombo F. (2018). Biofilms in the food industry: Health aspects and control methods. Front. Microbiol..

[25] Gafri H.F.S., Zuki F.M., Aroua M.K., Hashim N.A. (2019). Mechanism of bacterial adhesion on ultrafiltration membrane modified by natural antimicrobial polymers (Chitosan) and combination with activated carbon (PAC). Rev. Chem. Eng..

[26] Zhu J., Hou J., Zhang Y., Tian M., He T., Liu J., Chen V. (2018). Polymeric antimicrobial membranes enabled by nanomaterials for water treatment. J. Membr. Sci..

[27] Mukherjee M., De S. (2018). Antibacterial polymeric membranes: A short review. Environm. Sci. Water Res. Technol..

[28] Zhu Y., Wang J., Hou J., Zhang Y., Liu J., Ven der Bruggen B. (2017). Graphene-based antimicrobial polymeric membranes: A review. J. Mater. Sci. A.

[29] Akbari S., Kozlowski R.M. (2019). A review of application of amine-terminated dendritic materials in textile engineering. J. Text. Inst..

[30] Emam H.E. (2019). Antimicrobial cellulosic textiles based on organic compounds. 3 Biothech..

[31] Ul-Islam S., Butola B.S. (2019). Recent advances in chitosan polysaccharide and its derivatives in antimicrobial modification of textile materials. Int. J. Biol. Macromol..

[32] Morais D.S., Guedes R.M., Lopes M.A. (2016). Antimicrobial approaches for textiles: From research to market. Materials.

[33] Oblak E., Piecuch A., Rewak-Soroczynska A., Paluch E. (2019). Activity of gemini quaternary ammonium aalts against microorganisms. Appl. Microbiol. Biotechnol..

[34] Fait M.E., Bakas L., Garrote G.L., Morcelle S.R., Saparrat M.C.N. (2019). Cationic surfactants as antifungal agents. Appl. Microbiol. Biothechnol..

[35] Makvandi P., Jamaledin R., Jabbari M., Nikfarjam N., Borzacchiello A. (2018). Antibacterial quaternary ammonium compounds in dental materials: A systematic review. Dent. Mater..

[36] Mulder I., Siemens J., Sentek V., Amelung W., Smalla K., Jachalke S. (2018). Quaternary ammonium compounds in soil: Implications for antibiotic resistance development. Rev. Environ. Sci. Bio-Technol..

[37] Jiao Y., Niu L.-n., Ma S., Li J., Tay F.R., Chen J.-h. (2017). Quaternary ammonium-based biomedical materials: State-of-the-art, toxicological aspects and antimicrobial resistance. Progr. Polym. Sci..

[38] Jennings M.C., Minbiole K.P.C., Wuest W.M. (2015). Quaternary ammonium compounds: An antimicrobial mainstay and platform for innovation to address bacterial resistance. Acs Infect. Dis..

[39] Gerba C.P. (2015). Quaternary ammonium biocides: Efficacy in application. Appl. Environ. Microbiol..

[40] Buffet-Bataillon S., Tattevin P., Bonnaure-Mallet M., Jolivet-Gougeon A. (2012). Emergence of resistance to antibacterial agents: The role of quaternary ammonium compounds-a critical review. Int. J. Antimicrob. Agents.

[41] Yoo J.H. (2018). Review of disinfection and sterilization – Back to the basics. Infect Chemother..

[42] Inacio A.S., Domingues N.S., Nunes A., Martins P.T., Moreno M.J., Estronca L.M., Fernandes R., Moreno A.J.M., Borrego M.J., Gomes J.P., Vaz W.L.C., Vieira O.V. (2016). Quaternary ammonium surfactant structure determines selective toxicity towards bacteria: Mechanisms of action and clinical implications in antibacterial prophylaxis. J. Antimicrob. Chemother..

[43] Zubris D.L., Minbiole K.P.C., Wuest W.M. (2017). Polymeric quaternary ammonium compounds: Versatile antimicrobial materials. Curr. Top. Med. Chem..

[44] Zhang W., Zhou J.J., Dai X.L. (2017). Preparation and characterization of reactive chitosan quaternary ammonium salt and its application in antibacterial finishing of cotton fabric. Text. Res. J..

[45] Xue Y., Xiao H.N., Zhang Y. (2015). Antimicrobial polymeric materials with quaternary ammonium and phosphonium salts. Int. J. Mol. Sci..

[46] Mancuso R., Amuso R., Armentano B., Grasso G., Rago V., Cappello A.R., Galiano F., Figoli A., De Luca G., Hoinkis J., Gabriele B. (2017). Synthesis and antibacterial activity of plymerizable acryloyloxytriethyl ammonium salts. ChemPlusChem.

[47] Deowan S.A., Galiano F., Hoinkis J., Johnson D., Altinkaya S.A., Gabriele B., Hilal N., Drioli E., Figoli A. (2016). Novel low-fouling membrane bioreactor (MBR) for industrial wastewater treatment. J. Membr. Sci..

[48] Galiano F., Figoli A., Deowan S.A., Johnson D., Altinkaya S.A., Veltri L., De Luca G., Mancuso R., Hilal N., Gabriele B., Hoinkis J. (2015). A step forward to a more efficient wastewater treatment by membrane surface modification via polymerizable biocontinuous microemulsion. J. Membr. Sci..

[49] Galiano F., Schmidt S.A., Ye X., Kumar R., Mancuso R., Curcio E., Gabriele B., Hoinkis J., Figoli A. (2018). UV-LED induced bicontinuous microemulsion polymerisation for surface modification of commercial membranes – Enhancing the antifouling properties. Sep. Pur. Technol..

[50] De Luca G., Amuso R., Figoli A., Mancuso R., Lucadamo L., Gabriele B. (2018). Modeling of structure-property relashionships of polymerizable surfactants with antimicrobial activity. Appl. Sci..

[51] Tomar N., Tomar M., Gulati N., Nagaich U. (2012). pHEMA hydrogels: Devices for ocular drug delivery. Int. J. Heal. Allied Sci..

[52] Silhavy T.J., Kahne D., Walker S. (2010). The bacterial cell envelope. Cold Spring Harb. Perspect. Biol..

[53] Sundararaman M., Kumar R.R., Venkatesan P., Ilangovan A. (2013). 1-Alkyl-(N,N-dimethylamino)pyridinium bromides: Inhibitory effect on virulence factors of Candida Albicans and on the growth of bacterial pathogens. J. Med. Microbiol..

[54] He J., Söderling E., Österblad M., Vallittu P.K., Lassila L.V.J. (2011). Synthesis of methacrylate monomers with antibacterial effects against S. mutans. Molecules.

[55] Sezonov G., Joseleau-Petit D., D’Ari R. (2007). Escherichia Coli physiology in Luria-Bertani broth. J. Bacteriol..

